# Optical nonlinearities in ultra-silicon-rich nitride characterized using z-scan measurements

**DOI:** 10.1038/s41598-019-46865-7

**Published:** 2019-07-17

**Authors:** Byoung-Uk Sohn, Ju Won Choi, Doris K. T. Ng, Dawn T. H. Tan

**Affiliations:** 1 Photonics Devices and System Group, Engineering Product Development, Singapore University of Technology and Design, 487372 Singapore, Singapore; 2 Institute of Microelectronics, A*STAR (Agency for Science, Technology and Research), 2 Fusionopolis Way, #08-02, Innovis Tower, 138634 Singapore, Singapore

**Keywords:** Nonlinear optics, Nonlinear optics

## Abstract

The dispersive nonlinear refractive index of ultra-silicon-rich nitride, and its two-photon and three-photon absorption coefficients are measured in the wavelength range between 0.8 µm–1.6 µm, covering the O- to L – telecommunications bands. In the two-photon absorption range, the measured nonlinear coefficients are compared to theoretically calculated values with a simple parabolic band structure. Two-photon absorption is observed to exist only at wavelengths lower than 1.2 μm. The criterion for all-optical switching through the material is investigated and it is shown that ultra-silicon-rich nitride is a good material in the three-photon absorption region, which spans the entire O- to L- telecommunications bands.

## Introduction

Ultra-silicon-rich nitride (USRN), with composition Si_7_N_3_, is a promising platform for optical signal processing at the telecommunications wavelengths because of its large nonlinear refractive index and absence of two-photon absorption. The refractive index and band gap of the silicon-rich nitride material can be controlled by the silicon content^1^. A larger silicon content decreases the energy band gap, towards that of silicon (1.11 eV). In silicon, strong two-photon absorption exists in the entire telecommunications band, which makes it less efficient for nonlinear optical processes. The ratio of silicon to nitrogen content can be engineered in order to tailor the bandgap. USRN has been successfully used to demonstrate high gain optical parametric amplification, four-wave mixing and photonic crystal waveguides^1–10^. A wide band gap reduces not only absorption loss but also the nonlinear refractive index; There exists a tradeoff between high nonlinear refractive index and low absorption loss. This tradeoff arises as a result of Kramers-Krönig (K-K) relations that govern the relationship between nonlinear refractive index and multi-photon absorption. USRN’s larger bandgap allows two-photon absorption to be negligible at the 1.55 μm wavelength. A further advantage of USRN’s larger bandgap pertains to its optical transparency at shorter wavelengths than that in silicon – USRN is optically transparent at wavelengths as low as 0.6 μm compared to 1.1 μm in silicon.

In this paper, we characterize the nonlinear properties of USRN using the z-scan method across a broad range of wavelengths between 0.8 μm to 1.6 μm. We quantify the two- and three-photon absorption coefficients of the USRN platform which possesses a band gap of 2.1 eV^4,6^ and show that two-photon absorption becomes non-negligible when the wavelength drops below 1.2 μm. Since the USRN films are grown using chemical vapor deposition, the film thicknesses are limited. Consequently, the z-scan measurements will be limited by the measurable intensity dip during the z-scan measurements. Even with these limitations, we were able to characterize the three-photon absorption coefficients up to a wavelength of 1.6 μm.

## Results

USRN films with a thickness of 1.1 μm were deposited on a 50 μm thick SiO_2_ substrate using inductively coupled chemical vapor deposition^6^ at relatively low temperature of 250 °C. In the deposition, N_2_ gas is used in replace of NH_3_ for chemical reaction in forming Si_7_N_3_ to minimize amount of H in the film because Si-H or N-H is a dominant absorption loss bonding at communication wavelength range of 1510–1565 nm^11–15^. The material composition was previously characterized using FTIR spectroscopy. The FTIR measurements did not pick up the characteristic absorption peak from Si-H bonds close to 1550 nm. Therefore, hydrogen content in the film if present, is below the FTIR detection limit and should be relatively low.

**Figure 1:**
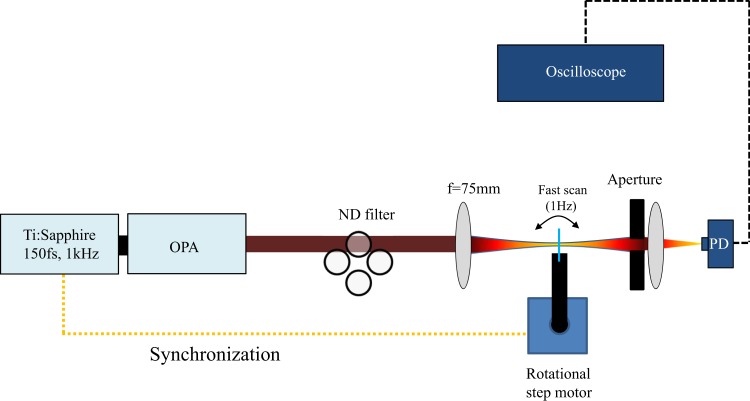
Schematic diagram of the z-scan setup. OPA refers to optical parametric amplifier, ND stands for neutral density and PD refers to photodetector.

We employ the z-scan method to characterize the nonlinear coefficient of the USRN; The thin sample thickness places a very high signal to noise ratio requirement to retrieve nonlinear coefficients from very small valley-peak in the measured signal. For this purpose, we formulated a very fast z-scan as shown in Fig. 1 to acquire a large number of averages and in so forth, minimize any contributions to noise. The sample is set at the end of a long arm that is connected to a rotational step motor. The IR ranges of the optical pulses used in the experiment are derived from an optical parametric amplifier (OPA). The OPA is pumped using a Ti:Sapphire laser producing 150 fs pulses at 800 nm at a repetition of 1 kHz. To make many measurements averaged in the same condition, we synchronize the rotational step motor with the laser. Since the sample stage is moving at a specific frequency of 1 Hz and the laser repetition rate is 1 kHz, it is possible to synchronize both frequencies such that all pulses from the laser are incident at the same point on the sample. Without proper synchronization, measurements with each subsequent pulse will have a different z-scan distribution and averaging will not be effective. The laser power has a 10% fluctuation. Consequently, averaging with a large number of measurements (*N*) is used to reduce the resulting noise. The laser fluctuation scales according to $10 \% /\sqrt{N}$ by random walk distribution in which a standard deviation proportional to $\sqrt{N}$ is divided by an average proportional to *N*. Consequently, the peak-valley difference in the z-scan measurements may be more easily resolved with averaging applied.

Using the aforementioned approach, power fluctuations in the laser (~10%) were successfully reduced to less than 0.3% over a thousand averages. The arm length was designed to be almost 30 cm long to ensure parallel movement and fast scanning with the rotational motor.

The USRN films are grown on a thin SiO_2_ substrate (50 μm) to minimize unnecessary effects from the substrate. The n_2_ value for SiO_2_ is well known to be low (2.7 × 10^−20^ m^2^/W at 1.55um). The effect of the substrate is accounted for in the z-scan measurements. We considered field distortion by multi-films especially two-photon absorption for the simplicity of mathematics. It can be generalized to a higher order of nonlinear absorption. The effect of material on the field going through the sample is described by two coupled equations. $$d\Delta \varphi /dz^{\prime} =2\pi {n}_{2}I/\lambda $$ and $$dI/dz^{\prime} =-{\beta }_{2}{I}^{2}$$, where $$z^{\prime} $$ is the sample coordinate, $$\Delta \varphi $$ is nonlinear phase distortion by the nonlinear sample, *n*_2_ is the nonlinear refractive index and *β*_2_ is the two photon absorption coefficient. The field distribution at the sample output can be obtained analytically, $${E}_{e}={E}_{in}(z,r,t){(1+{\beta }_{2}L{I}_{in})}^{ik{n}_{2}/\beta -1/2}$$. For small multi-photon absorption coefficient approximation, $${\beta }_{2}L{I}_{in}$$ ≪ 1, $${E}_{e}={E}_{in}(z,r,t){{\rm{e}}}^{ik{n}_{2}L{I}_{in}}{{\rm{e}}}^{-{\beta }_{2}L{I}_{in}/2}$$. In this approximation, the multi-film effect is additive because the nonlinear coefficients are located the argument of the exponential function. The measured nonlinear coefficients have some contribution of the substrate as follows, $${n}_{2}+{{n}_{2}}^{^{\prime} }(1-{(\frac{n-n^{\prime} }{n+n^{\prime} })}^{2})L^{\prime} /L$$ and $${\beta }_{2}+{{\beta }_{2}}^{^{\prime} }(1-{(\frac{n-n^{\prime} }{n+n^{\prime} })}^{2})L^{\prime} /L$$. For n-photon absorption, the expression can be generalized to $${\beta }_{n}+{{\beta }_{n}}^{^{\prime} }$$$${(1-{(\frac{n-n^{\prime} }{n+n^{\prime} })}^{2})}^{n-2}L^{\prime} /L$$, where the term $${(\frac{n-n^{\prime} }{n+n^{\prime} })}^{2}$$ denotes the Fresnel reflection loss in the substrate.

**Figure 2:**
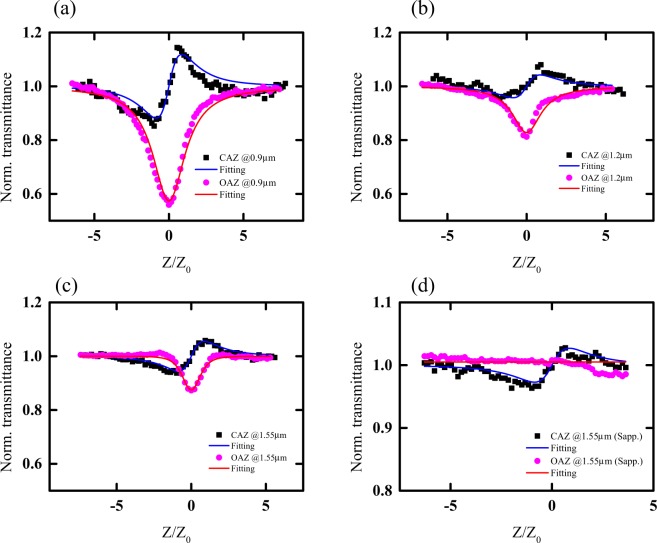
Measured z-scans of the USRN at the wavelength of (**a**) 0.9 µm, (**b**) 1.2 µm, and (**c**) 1.55 µm and (**d**) the Sapphire crystal at 1.55 μm.

The measured closed and open aperture z-scan of the thin USRN sample at three representative wavelengths are shown in Fig. 2. At 1.55 μm, the normalized intensity dips by 0.1 – a small value which can still be clearly resolved with the z-scan setup. The nonlinear absorption coefficients are retrieved by fitting the measured open aperture z-scan data with the expression, ${T}_{nPA}=1/{\{1+(N-1){\alpha }_{N}{L}_{eff}^{(N)}{[{I}_{00}/(1+{(z/{z}_{0})}^{2})]}^{N-1}\}}^{1/(N-1)}$^16,17^, where *a*_N_ is the absorption coefficient, *N* is the contributed number of photons, ${I}_{00}$ is the peak intensity of the beam at the focal point, ${L}_{eff}^{(N)}$ is the effective thickness of sample and *z*_0_ is Rayleigh length. Closed aperture z-scan data is normalized by open aperture z-scan data to remove nonlinear absorption effects before fitting with the expression, $T=1+\frac{4{\rm{\Delta }}\varphi z/{z}_{0}}{[1+{(z/{z}_{0})}^{2}][9+{(z/{z}_{0})}^{2}]}$. In the expression for *T*, ${\rm{\Delta }}\varphi =2\pi {I}_{00}{L}_{eff}{n}_{2}/\lambda$ is the defined nonlinear phase generated by the nonlinear refractive index. Our z-scan system is calibrated with sapphire glass. The measured z-scan for sapphire glass is shown in Fig. 2(d). The retrieved value by the system was 4.2 × 10^−20^ m^2^/W at 1.55 μm, a comparable value to that reported ref.^18^ (2.8 × 10^−20^m^2^/W). Thus, we scaled our measured values by a factor of 0.66 to account for the variation in the measured *n*_2_ from laser peak intensity used in the experiments.

**Figure 3:**
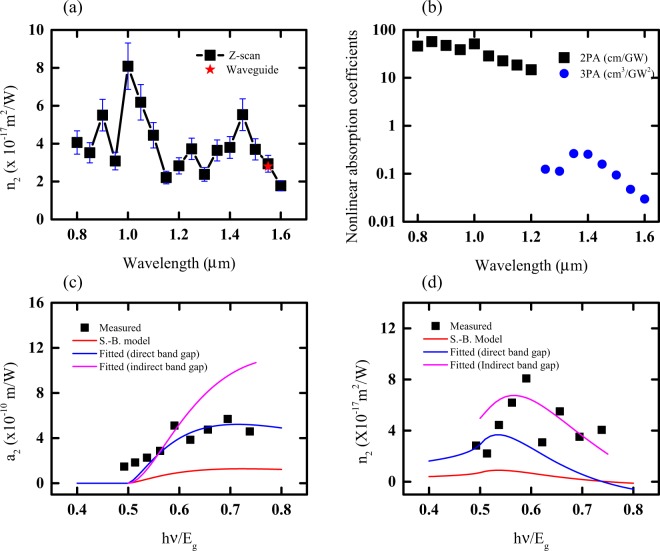
(**a**) The nonlinear refractive index measured using z-scan (black squares) and USRN waveguide experiments (red star)^4^. (**b**) Multi-photon absorption coefficients of USRN in the IR range characterized using z-scan measurements. Measured and theoretical values of (**c**) two-photon absorption coefficients and (**d**) the Kerr nonlinear refractive index are shown. The red solid line represents the theoretical calculation in a direct band gap Sheik-Bahae (S.-B.) model; The blue lines are the fitted result by changing the K value in the Sheik-Bahae model, and magenta lines are the fitted results with the model for indirect band materials.

The measured dispersive refractive indices between 0.8 μm to 1.6 μm are shown in Fig. 3(a). At 1.55 μm, the measured *n*_2_ is 2.93 × 10^−17^ m^2^/W, a value which is similar to that which was previously measured using self-phase modulation experiments^4^ as drawn with a red star point in Fig. 3(a). It is observed that the peak *n*_2_ value of 8.1 × 10^−17^m^2^/W occurs at 1 µm, close to the two-photon edge (*E*_g_ = hc/*λ* ~ 0.6). This is consistent with K-K relations, that describes the nonlinear refractive index to possess its highest value near the two-photon wavelength, as described by the expression, ${n}_{2}(\omega ;{\rm{\Omega }})=\frac{{\rm{c}}}{{\rm{\pi }}}{\int }_{0}^{\infty }\frac{{\alpha }_{2}(\omega ^{\prime} ,\Omega )}{{\omega ^{\prime} }^{2}-{\omega }^{2}}d\omega ^{\prime}$. Further, K-K relations predict a plateauing in *n*_2_ value at longer wavelengths. Despite this prediction, we observe an additional peak in *n*_2_ near 1.45 µm. We hypothesize that this peak arises from an effect of higher nonlinear refractive index (*n*_4_) beyond the Kerr nonlinear refractive index (*n*_2_). The *n*_4_ is related to the three-photon absorption coefficient by K-K relation, ${n}_{4}(\omega ;\Omega ,\Omega ^{\prime} )=\frac{{\rm{c}}}{{\rm{\pi }}}{\int }_{0}^{\infty }\frac{{\alpha }_{3}(\omega ^{\prime} ,\Omega ,\Omega ^{\prime} )}{{\omega ^{\prime} }^{2}-{\omega }^{2}}d\omega ^{\prime}$, where ${n}_{4}(\omega ;\Omega ,\Omega ^{\prime} )$ and ${\alpha }_{3}(\omega ,\Omega ,\Omega ^{\prime} )$ denote respectively, a non-degenerate nonlinear index and an absorption at the existence of photons of *ω*, Ω and $\Omega ^{\prime}$ frequency. The peak of three-photon absorption appears near 1.45 µm (0.4E_g_) as shown in Fig. 3(b). At wavelengths larger than 1.2 μm, two-photon absorption vanishes and the dominant nonlinear loss mechanism is three-photon absorption. The three-photon absorption coefficient is on the order of 10^−25^ m^3^W^−2^ within the measurement range between 1.2 μm to 1.6 μm. Nonlinear losses in this region are therefore characterized to be very small in the USRN film – a highly advantageous feature for nonlinear optics applications.

Some research groups have calculated the nonlinear refractive index and two-photon absorption coefficient based on K-K relations in which a direct bandgap or indirect band gap having a simple parabolic band structure have been used. Sheik-Bahae *et al.*^19^ have calculated the nonlinear refractive index and the two-photon absorption as well described in a parabolic band structure. The degenerate two-photon absorption at a direct band gap value, *E*_*g*_ is given by ${\alpha }_{2}(\omega )=K\frac{\sqrt{{E}_{p}}}{{n}_{0}^{2}{E}_{g}^{3}}{F}_{2}(\frac{\hslash \omega }{{E}_{g}})$, where the function, ${F}_{2}(x)={(2x-1)}^{3/2}/{(2x)}^{5}$ reflects the band structure and intermediate states, *K* = 1900~5200 is a model dependent constant, *E*_*p*_ = 2|p_vc_|^2^/m_0_ ~ 21 eV for direct band gap semiconductors, *n*_0_ is a linear refractive index and *E*_*g*_ is the band gap energy. The nonlinear refractive index is calculated from the K-K relation by assuming 2ω → ω + Ω and Ω = ω is set after the integral calculation. This assumption is valid for the case of degenerate nonlinear refractive indices. Consequently, the nonlinear refractive index is calculated as ${n}_{2}(\omega )=K\frac{\sqrt{{E}_{p}}}{{n}_{0}^{2}{E}_{g}^{3}}\frac{\hslash c}{\pi {E}_{g}}\mathrm{Re}{\int }_{0}^{\infty }dx\frac{{x}^{3/2}}{{(x+1)}^{6}(x-i\gamma +1-2\hslash \omega /{E}_{g})}$, where we inserted a small quantity, γ ≪ 1, which represents a phenomenological damping loss to avoid singularities in the integral calculation. The calculated theoretical values are drawn Fig. 3(c,d). The red lines in the figure correspond to expected values from Sheik-Bahae model at K = 5200. The expected values are smaller than our measured nonlinear coefficient values. The blue lines are fitted for two-photon absorption coefficients from the Sheik-Bahae model at K = 20000. For this value of K, the nonlinear refractive index is not well fitted. The deviation in the dispersive shape reflects that the sample’s band diagram is different from the simple parabolic band structure. In Fig. 3(c), the measured two-photon absorption coefficient near the band edge does not vanish contrary to theoretical predictions that the value should drop to zero. This phenomenon likely arises from the existence of edge states and localized defects induced by broken random symmetry in the amorphous USRN material. This phenomenon gives rise to band tail states, also known as Urbach tails^20^. Furthermore, the measured peak *n*_2_ location is slightly blue-shifted compared to theoretical predictions.

The blue-shift observed in the peak *n*_2_ location may be overcome by introducing an indirect band structure. The two-photon absorption coefficient and the nonlinear refractive index have previously been calculated in an indirect bandgap material^21,22^. The electronic transition in the indirect bandgap needs phonon-electron interactions to satisfy momentum conservation, particularly since the momentum of a photon is negligibly small. The two-photon absorption process needs a virtual state as an intermediate state to make the transition from the valence band to the conduction band. A forbidden or an allowed transition is possible between the states depending on parity. Thus, the two-photon absorption process has three types of possible transitions: (i) allowed-allowed, (ii) allowed-forbidden, and (iii) forbidden-forbidden transitions^22^. The amplitude of the momentum operator of electrons that transition from the valence band to the conduction band (virtual states) varies by parity. The square of the transition amplitude is proportional to *E*_*g*_ for the allowed transition and proportional to (2ħ*ω*-*E*_*g*_) for the forbidden transition. The two-photon absorption coefficient has the functional form, ${\alpha }_{2}=C\frac{{(2x-1)}^{2+n}}{{(2x)}^{5}}$, where C is the parameter containing phonon effect defined by $C=\frac{{m}_{\mu }^{3/2}\sqrt{{E}_{p}}K}{4\sqrt{2}{\pi }^{2}{\hslash }^{3}{n}_{0}^{2}{E}_{g}^{3/2}}{|{H}_{ep}|}^{2}$(*m*_*μ*_ is an effective mass of electron-hole system and *H*_*eP*_ is an electron-phonon matrix), *x* is ħ*ω*/*E*_*g*_ and *n* = 0, 1, 2 for allowed-allowed, allowed-forbidden, and forbidden-forbidden transitions respectively. In this paper, the functions are used to compare with our experimental results as shown in Fig. 3(c,d). The magenta lines in the figure are for the measured nonlinear refractive values fitted with the indirect band gap model. The allowed-allowed transitions dominant near the two-photon edge is used for the fit. The peak position of the refractive index is well matched to the experimental results for the transition calculation in the indirect bandgap. However, with the fitted value for nonlinear refractive value, the two-photon absorption graph was not satisfied as shown in Fig. 3(c). Consequently, for theory and experiment of dispersive nonlinear optical properties in the USRN to have perfect agreement, detailed information pertaining to the band structure and existing localized band states is needed. In the indirect band gap model, the nonlinear parameters are related to the electron-phonon matrix. Therefore, it follows that the characterized nonlinear coefficients could be used for indirect measurements of the electron-photon matrix, which is important to understand the interaction between electrons and phonons.

It is noted that the measured nonlinear refractive index of the USRN film is higher than that in crystalline silicon. Some groups have reported that deposited amorphous Si:H has a higher nonlinear refractive than crystalline Si^23,24^. The higher nonlinear refractive index arises from a free carrier effect excited by two step absorption in the existence of defect states in amorphous-Si^23^. The nonlinear refractive index has the same sign as the Kerr nonlinear index^24^. We postulate that the same phenomenon could explain the high nonlinear refractive index in USRN films. Although more systematic explanation of the origin of the high nonlinearity is needed, it is possible that the increased nonlinear refractive index observed in USRN relative to crystalline silicon could arise from a single photon resonance from band states located in the half-band gap. The 3^rd^ order nonlinear susceptibility tensor in the randomly homogeneous amorphous systems has two independent components, the two-photon process and one-photon process. The one-photon process is usually negligible and ignored for wide band gaps. However, the one-photon process could be important in band gap states generated by defects in the amorphous USRN material. For the nonlinear refractive index, the susceptibility ${\chi }_{1111}(\omega ;\omega -\omega +\omega )$ corresponding to the one photon process is proportional to $\frac{1}{{({E}_{b}-{E}_{v}-\hslash \omega )}^{2}+{\gamma }^{2}}\frac{1}{{E}_{b}-{E}_{v}-\hslash \omega +i\gamma }$, where *E*_*b*_, *E*_*v*_ and *ℏω* refer to the energy of band states, the valence band, and the photon respectively. The vanishing denominator term contributes to the resonant increase of nonlinear refractive index in the existence of band states. These defect states potentially give rise to an increase in the nonlinear refractive index.

**Figure 4:**
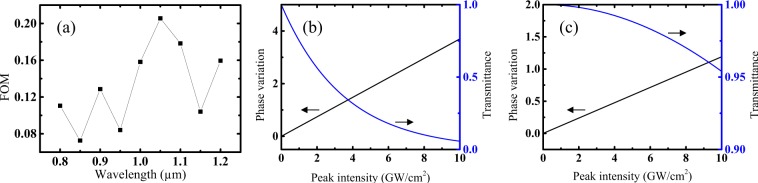
(**a**) FOM for the USRN material within the two-photon absorption region. (**b**) The achievable phase variation and two-photon absorption loss vs. peak intensity at 1.05 µm based on the measured nonlinear coefficients. (**c**) The achievable phase variation and three-photon absorption loss vs. peak intensity at 1.55 µm.

Nonlinear absorption gives rise to unwanted attenuation in signal processing applications^25–28^. For all-optical optical switching devices leveraging the nonlinear refractive index, a tradeoff exists between high nonlinear refractive index and low nonlinear absorption. The nonlinear phase variation is achieved according to ${n}_{2}kIL$, where *k* is the wave number, *I* is peak intensity and *L* is a propagation length of a beam through a nonlinear material. The nonlinear absorption loss is governed by ${\alpha }_{2}IL$. The nonlinear figure of merit (FOM) defined as n_2_/λα_2_, is a commonly used quantity in the two-photon absorption region for assessing the suitability of a material for all-optical switching applications. For the directional coupler possessing the most stringent requirements, FOM > 2 should be satisfied^29^. The calculated FOM for USRN in the two-photon absorption region (*λ* < 1.2 μm) is less than 0.2, not satisfying the criterion for switching application as shown in Fig. 4(a). The achievable phase variation by the nonlinear effect and signal loss by the nonlinear absorption at 1.05 µm is drawn in Fig. 4(b), where the propagation length is assumed to be 100 µm. The figure implies that the energy of the input beam is largely absorbed before the required phase variation for a switching application is achieved. However, for longer wavelengths larger than 1.2 µm within the three-photon absorption region, switching applications may be efficiently implemented as shown in Fig. 4(c). The telecommunications wavelength of 1.55 µm, and a propagation length of 100 µm is used. Using a technique similar to that used for the two-photon absorption region, the phase variation can be calculated according to ${n}_{2}k\cdot I\cdot L$ and nonlinear absorption loss is calculated using ${\alpha }_{3}{I}^{2}L$. The phase variation is 1.75 at the 10 GW/cm^2^ peak intensity although the three-photon nonlinear absorption loss is just 7%. Greater phase variation can be obtained if the requirement on the absolute amount of nonlinear loss is relaxed. Consequently, the USRN material is a good material for all-optical switching in the complete O- to L- telecommunications bands because of the high nonlinear refractive index and the small three-photon absorption coefficient.

## Conclusions

We have experimentally characterized the dispersive nonlinear refractive index, two- and three-photon absorption coefficients at wavelengths between 0.8µm–1.6 µm. Between 0.8µm–1.2 µm, two-photon absorption is observed and the trends for the nonlinear refractive index and two-photon absorption are observed to satisfy K-K relations. The measured coefficients are comparable to the theoretically calculated values based on K-K relations. The measured spectral form is different from the calculation based on a simple band structure assuming a parabolic form. This implies that the difference observed in the measured spectral form could be used to retrieve a complex band structure. The resonant peak having the highest value, 8.1 × 10^−17^ m^2^/W, exists at 1 µm corresponding to the two-absorption edge. The FOM in the two-photon absorption region is too small to satisfy the criterion for all-optical switching. Fortunately, the USRN material is demonstrated to have a vanishing two-photon absorption at wavelengths beyond 1.2 μm, where telecommunications applications operate. In the three-photon absorption region, 1.2 µm–1.6 µm, covering the complete O- to L- telecommunications bands, the nonlinear refractive index is large and the three-photon absorption is small, making USRN highly advantageous for nonlinear optics in this region. The criterion for all-optical switching in this region is easily satisfied. Based on the measured dispersive nonlinear coefficients of the USRN, a proper propagation lengths and intensity can be selected to design low power, all-optical switching devices or modulators at optical communication wavelengths. The results further show USRN to be an advantageous material for nonlinear optics applications at the telecommunications wavelengths.
